# The Work of the Holy Ghost

**Published:** 1887-07-02

**Authors:** 


### Words of Consolation.

[Communications for this column should be addressed, " Chaplain," care of the Editor of The Hospital, who will gratefully welcome any suggestions or inquiries from matrons, nurses, and the staff generally.]

### XXXIX.—The Work of the Holy Ghost.

The Bible and the Church are the two chief witnesses from without, through which the Holy Ghost speaks to us. Conscience is the witness within, whose testimony should agree to the teaching of the other two. When, therefore, we find nominal Christians with consciences excusing them from obedience to some of the plainest precepts, or most sacred ordinances, which have been considered binding by really conscientious people everywhere, and at all times, we can only conclude that such consciences are blinded, and therefore misguided. Of course we all receive rebukes through conscience; but these belong rather to convictions than to guidance. Wherever the guidance of the Spirit is truly manifested in conscience, the three witnesses will be found in agreement upon all first principles, upon all the essentials of salvation. But if we pray earnestly for the Spirit of Counsel, the guidance of the Holy Ghost is vouchsafed to keep us, not only concerning things which are lawful or unlawful, but in many questions of expediency as well. " All things are lawful unto me: but all things are not expedient" (1 Cor. vi, 12). In the life of penitence questions arise far more often over what is expedient or the reverse, than over what is lawful. How, then, may we know when to trust conscience? When may we suppose the voice to be the voice of God, and therefore right, and when not? One condition of first importance is that penitence must be real. There must be no sort of resistance to the motions of the Spirit. We may be quite sure that conscience is not to be trusted if either mind or body are slaves to any one besetting sin. This is quite enough to blind conscience, not totally perhaps, but quite sufficiently for it to forfeit any claim to true guidance. You know if a compass is in good order the needle will always point in one direction, to the north. If the compass is disordered the needle may point anywhere; you cannot rely upon it. So with a spiritual being disordered by a habit of sin: the needle of conscience may keep pointing again and again in every direction but the way most needed. And why? because sin quenches the Spirit; and though He may long continue to rebuke or plead, He is too far dethroned to act as ruler or guide. On the other hand, just in proportion as the whole life is pure and free from low, sordid, and selfish motives, so is the certainty of the Spirit's guidance to be counted on. " The meek will be the guide in judgment" (Ps. xxv, 9). " The path of the just is as the shining light" (Prov. iv, 18). The pure in heart *see* as no others do. These are the ones to whom is granted " a right judgment in all things."

(*To be continued.*)

